# Crocin protects against smoke-induced chronic obstructive pulmonary disease by regulating AKT1

**DOI:** 10.3389/fphar.2026.1687752

**Published:** 2026-02-09

**Authors:** Yuehong Zhu, Yuntao Jiang, Jieping Xu, Sicheng Yan, Zhihong Ma

**Affiliations:** 1 Huzhou Central Hospital, Fifth School of Clinical Medicine of Zhejiang Chinese Medical University, Huzhou, China; 2 Huzhou Central Hospital, Affiliated Central Hospital of Huzhou University, Huzhou, China; 3 Deqing People’s Hospital, Huzhou, China

**Keywords:** anti-inflammation, COPD, crocin, molecular dynamics, multi-target, network analysis, Pi3k-akt

## Abstract

**Background:**

Chronic obstructive pulmonary disease (COPD) is an inflammatory airway disorder characterized by persistent airflow limitation and pathological features such as airway remodeling. Identifying molecular targets involved in airway epithelial dysfunction is crucial for developing COPD therapies. Crocin, a carotenoid glycoside from saffron (*Crocus sativus* L.), may exhibit pan-assay interference compounds (PAINS)-like properties owing to its conjugated polyene structure. This can lead to non-specific effects *in vitro* and complicate its pharmacological interpretation. Therefore, a multidimensional assessment strategy (network analysis + *in vitro* + *in vivo*) is essential to mitigate such limitations.

**Methods:**

We first employed predictive strategies, including network analysis, to identify common targets of crocin and COPD. Protein-protein interaction (PPI) networks were constructed, and core targets were screened via topology analysis. Gene Ontology (GO) and Kyoto Encyclopedia of Genes and Genomes (KEGG) enrichment analyses were performed to predict signaling pathways. Molecular docking and dynamics simulations were then used to assess the binding potential between crocin and the core targets. Finally, the function of crocin against COPD was evaluated using the Cellular Thermal Shift Assay (CETSA) *in vitro* and a cigarette smoke-induced mouse model *in vivo*.

**Results:**

Network analysis predicted 243 common targets, from which 48 candidate targets were identified. GO and KEGG enrichment analyses suggested the PI3K-AKT signaling pathway as a potentially key mechanism. Among the top-ranked core targets, molecular docking indicated favorable binding energies between crocin and proteins such as ALB and AKT1, a finding further corroborated by molecular dynamics simulations. Subsequent CETSA suggested a direct interaction between crocin and AKT1. *In vivo* experiments demonstrated that crocin administration significantly alleviated lung injury and inflammation and reduced the p-AKT1/AKT1 ratio, consistent with network analysis and CETSA findings, suggesting the observed effects were not solely attributable to PAINS interference.

**Conclusion:**

These findings support the therapeutic potential of crocin in COPD through its anti-inflammatory activity and regulation of AKT1. Despite potential PAINS properties, the consistency across network, *in vitro*, and *in vivo* data strengthens the biological relevance of its observed effects.

## 1 Introduction

Chronic obstructive pulmonary disease (COPD) is a complex inflammatory lung disorder characterized by persistent airway inflammation and irreversible airflow limitation (Xu et al., 2024). It is the third leading cause of global mortality, with projections indicating a significant rise in associated deaths by 2040 (Leung et al., 2019; Kahnert et al., 2023). The pathogenesis of COPD is driven by exposure to harmful particles (e.g., cigarette smoke), leading to airway remodeling, alveolar destruction, and chronic inflammation (Lin H. et al., 2022). Current treatments, including bronchodilators and glucocorticoids, provide symptomatic relief but fail to halt disease progression and carry risks of adverse effects such as osteoporosis with long-term use (Wang et al., 2020). This underscores the need for safer, multi-target therapies, positioning natural products like crocin as promising candidates.

Crocin, a water-soluble carotenoid from saffron (*Crocus sativus* L.), contains multiple adjacent phenolic hydroxyl groups. This structure confers Pan-assay interference compounds (PAINS)-like behavior, including nonspecific binding to media proteins (e.g., albumin), interference with redox-sensitive assays (e.g., ROS detection), and fluorescence quenching—all potentially masking true biological effects or causing false positives (Bolz et al., 2021; Magalhães et al., 2021).

Although crocin can modulate signaling pathways and molecular targets involved in inflammation and oxidative stress, and shows anti-inflammatory and antioxidant properties relevant to COPD (Pourmousavi et al., 2024; Wu et al., 2025), its PAINS characteristics complicate the interpretation of results from cell-based experiments. For instance, its apparent inhibition of NF-κB signaling may be due to nonspecific protein binding rather than specific pathway modulation (Du et al., 2018; Zhang et al., 2021). Similarly, its effects on oxidative stress and apoptosis in assays may reflect chemical interference with test reagents rather than true cellular activity. Therefore, conclusions based only on *in vitro* data may overestimate the actual pharmacological potential of crocin.

Despite these challenges, the therapeutic promise of crocin in COPD remains compelling. Capuzzi et al. noted that 87 FDA-approved small molecule drugs carry PAINS-related alerts, suggesting that such properties do not necessarily preclude clinical relevance (Capuzzi et al., 2017). Recent preclinical evidence further supports the potential of crocin administration. Specifically, Ghobadi et al. reported that crocin supplementation helps restore oxidant/antioxidant balance and ameliorate inflammatory markers in COPD patients (Ghobadi et al., 2022). Additionally, Aslani et al. found that crocin supplementation improves lung function parameters, enhances exercise tolerance, and reduces systemic inflammatory factors in individuals with COPD (Aslani et al., 2023). While these studies suggest crocin’s potential in COPD, their reliance on *in vitro* or single-model systems limits their ability to address PAINS-related concerns. To rigorously assess its biological relevance, multidimensional assessment—combining *in vitro* mechanistic studies with *in vivo* models—remains essential.

In this study, we provide a novel perspective on crocin-based therapeutic strategies for COPD by integrating network analysis, *in vitro* CETSA assays, and *in vivo* animal models. Although computational predictions of interactions between crocin and COPD-related targets/pathways are subject to inherent false-positive limitations, we mitigated this by sequentially combining target screening, molecular docking/dynamics simulations, and functional assessments in both cellular and mouse COPD models. This multi-layered approach overcomes the constraints of single-method studies and yields more robust insights into crocin’s therapeutic mechanisms in COPD.

## 2 Materials and methods

### 2.1 Network analysis

#### 2.1.1 Target gene analysis of crocin

**FIGURE 1:**
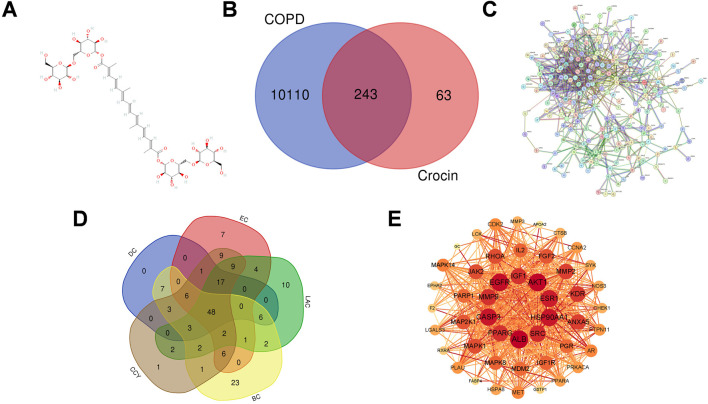
Network analysis of crocin in improving COPD. **(A)** Chemical structural formula of crocin. **(B)** Venn diagram of crocin target and COPD target. **(C)** PPI network analysis diagram constructed using STRING database. **(D)** PPI diagram of 48 candidate targets. **(E)** Screened using five dimensions of CytoNCA plug-in (DC, EC, LAC, BC, CCY) in Cytoscape, and the size and color of nodes correspond to the importance of each protein in the entire network. Red color and larger nodes indicate higher degree values. DC: Degree centrality, BC: Betweenness centrality, CCY: Closeness centrality, EC: Eigenvector centrality, LAC: Local average connectivity centrality.

The molecular structure of crocin was obtained from the PubChem database (https://pubchem.ncbi.nlm.nih.gov/; (Kim et al., 2025) (Figure 1A). Using the PharmMapper database (http://www.lilab-ecust.cn/pharmmapper/) and Similarity Ensemble Approach (SEA) (https://sea.bkslab.org) (Keiser et al., 2007), crocin target genes were obtained, combined and duplicates were screened to obtain the final target gene. In the PharmMapper (accessed 22 January 2025), targets were set to “Human Protein Targets Only” and the first 100 hits were taken as significant target proteins (Wang et al., 2017). We used the UniProt database (https://www.uniprot.org/, accessed 22 January 2025) to normalize the targets and obtain gene names corresponding to these targets. In the SEA database (accessed 22 January 2025), potential targets of crocin were obtained by searching and screening under conditions set to MaxTC >0.4.

#### 2.1.2 Target analysis of COPD

Using “chronic obstructive pulmonary disease” as the keyword, the GeneCards (https://www.genecards.org/accessed) (Stelzer et al., 2016), Online Mendelian Inheritance in Man (OMIM, https://omim.org/), and Therapeutic Target Database (TTD, http://db.idrblab.net/ttd/) (Zhou et al., 2024) database were searched for COPD disease-related genes, and duplicates were removed to yield the final list of COPD-related genes. The access date of the above databases was 22 January 2025.

#### 2.1.3 Protein–protein interaction network construction

Using an online website (https://bioinformatics.psb.ugent.be/webtools/Venn/), Venn diagrams of common targets of crocin and potential targets of COPD were plotted (Xie et al., 2024). Common targets were uploaded to the STRING database (https://cn.string-db.org) (Szklarczyk et al., 2023), organisms were limited to “*Homo sapiens*”, medium confidence was set at >0.7, isolated genes that did not interact with other genes were eliminated, and other parameters were set to default values. The network relationships among target proteins were then obtained. The topological properties of each node in the interaction network were then assessed by calculating the value of Degree centrality (DC), Betweenness centrality (BC), Closeness centrality (CCY), Eigenvector centrality (EC), and Local average connectivity centrality (LAC) using the Cytoscape3.10.1 CytoNCA plugin. Venn diagrams were drawn by selecting targets whose Degree, BC, CCY, EC, and LAC values were greater than the corresponding medians to obtain candidate targets for crocin in improving COPD (Duan et al., 2023). Finally, the candidate targets were analyzed by “Analyze Network” in Cytoscape3.10.1 software and PPI construction were performed according to Degree values.

#### 2.1.4 Gene Ontology (GO) and Kyoto Encyclopedia of Genes and Genomes (KEGG) pathway enrichment analysis

Through the SangerBox website (sangerbox.com/login.html) (Shen et al., 2022), GO and KEGG functional enrichment analysis of candidate targets was performed. Significantly enriched entries were those with *P-*value <0.05. The top 10 pathways as well as the top 10 terms in cellular component (CC), molecular function (MF), and biological process (BP) categories were selected to create bubble plots.

### 2.2 Molecular docking

The two-dimensional (2D) molecular structure of crocin was retrieved from PubChem database (https://pubchem.ncbi.nlm.nih.gov), downloaded, and converted into a three-dimensional (3D) structure using Chem3D 19.0 software. The crystal structures of key target proteins were acquired from the RCSB Protein Data Bank (https://www.rcsb.org/) (Iwasa et al., 2025). For each receptor protein, water molecules and small ligands were removed using PyMOL 3.0.4 to yield a clean receptor structure. Subsequently, hydrogen atoms were added to the protein, partial charges were assigned, and non-polar hydrogen atoms were merged using AutoDock Tools 1.5.6. Molecular docking simulations were carried out using AutoDock Vina 1.1.2. The docking box was constructed with the receptor protein at its center, fully enclosing the protein while the ligand was placed outside the box. Binding energy values indicate the likelihood of receptor–ligand interaction, with lower energies indicating stronger binding affinity and greater complex stability. Finally, the docking results were visualized using PyMOL 3.0.4.

### 2.3 Molecular dynamics simulation

In order to explore the stability of COPD-crocin interaction in more depth, molecular dynamics simulation (MD) was performed using the Gromacs 2022 program, GAFF force field for small molecules, AMBER14SB force field and TIP3P water model for proteins, and files of proteins and small molecule ligands were combined to construct a simulation system for the complex. Simulations were performed under constant temperature and pressure and periodic boundary conditions. During MD simulations, all involved hydrogen bonds were constrained using the LINCS algorithm with an integration step of 2 fs. Electrostatic interactions were calculated using the (Particle-mesh Ewald) PME method with a cutoff set at 1.2 nm. The nonbonded interaction cutoff was set to 10 Å and updated every 10 steps. The v-rescale temperature-coupling algorithm was used to maintain the temperature at 298 K and the Berendsen method was employed to control the pressure at 1 bar. At 298 K, 100 ps of NVT versus NPT equilibrium simulations were performed, and 100 ns of MD simulations were performed on the complex system, preserving the conformation every 10 ps. After the simulation was completed, the simulated trajectories were analyzed for root mean square deviation (RMSD), root mean square fluctuation (RMSF), radius of gyration (Rg), and number evolution of hydrogen bonds using VMD and pyMOL.

### 2.4 Cell Counting Kit-8 (CCK-8) assay

Cell viability was assessed using the CCK-8 (Beyotime, Cat# C0038) according to the manufacturer’s instructions. In brief, cells in the logarithmic growth phase were harvested and seeded into 96-well plates at a density of 5 × 10^3^ cells per well in 100 µL of complete medium. After 24 h of crocin treatment, 10 μL of CCK-8 solution was added directly to the culture medium. The plates were incubated for 2–4 h at 37 °C, and the optical density at 450 nm was recorded on a Thermo Scientific Varioskan LUX microplate reader. Cell viability was calculated as a percentage relative to the vehicle control group using the following formula: Viability (%) = [OD (treatment) - OD (blank)]/[OD (control) - OD (blank)] × 100. All experiments were performed with at least three independent replicates and each with five technical repeats.

### 2.5 Cellular Thermal Shift Assay (CETSA)

The Cellular Thermal Shift Assay (CETSA) was performed using human bronchial epithelial BEAS-2B cells. The cells were treated with 60 µM crocin or PBS as a vehicle control. After treatment, cells were harvested and resuspended in ice-cold PBS supplemented with protease and phosphatase inhibitors. The cell suspension was aliquoted and heated at various temperatures (37 °C–64 °C) for 3 min in a thermal cycler, followed by cooling on ice for 3 min. Cell lysis was achieved through three freeze-thaw cycles using liquid nitrogen. The soluble fraction was collected by centrifugation at 20,000 × g for 10 min at 4 °C. Protein concentration was determined using the BCA method, and Western blot analysis was subsequently performed with a target-specific antibody.

### 2.6 Experimental assessment of anti-inflammation effect of crocin in COPD

#### 2.6.1 Animals

Forty male, 6–8 weeks old SPF BALB/c mice were purchased from Zhejiang Vital River Laboratory Animal Technology Co., Ltd., with a body weight of 25 ± 2 g. All animals were maintained under standard conditions (humidity: 50% ± 5%, temperature: 22 °C ± 0.5 °C, 12 h light-dark cycle). Food and water were provided *ad libitum* during the feeding period, and experiments were conducted after a 7-day acclimatization period. The project was approved by the Experimental Animal Ethics Committee of Huzhou Central Hospital (Approval No. 202112002).

#### 2.6.2 Reagents

Crocin was purchased from Sigma-Aldrich (United States). Hongqi Canal cigarettes were purchased from Henan Anyang Cigarette Factory. TNF-α and IL-6 enzyme-linked immunosorbent assay (ELISA) kits were provided by Hangzhou Lianke Biotechnology Co., LTD. (Hangzhou, China). The antibodies used in the Western blot were purchased from Proteintech Group (Wuhan, China), with the following dilutions: AKT (1:2,000), phosphorylated AKT1 (1:1,500), and GAPDH (1:5,000).

#### 2.6.3 Animal model and treatment

COPD models in mice were established using a smoke-induced method vis a smoke exposure system (Shanghai Yuyan, Model C-350, Box 60 cm × 50 cm × 30 cm). (He et al., 2015; Liang and He, 2019; Lai et al., 2022). Briefly, after 7 days of adaptive feeding, 40 mice were divided into control, Model, Crocin-Low (Crocin-L), and Crocin-High (Crocin-H) groups using a random number table, with 10 mice in each group. The cigarettes we used each contained 10 mg of cigarette tar, 0.8 mg of nicotine, and 12 mg of carbon monoxide. Smoke induction was conducted 6 days a week, twice a day, with an interval of 4 h 10 cigarettes were used each time for 30 min of induction, and sealed for 1 h after smoke generation. After each smoking induction, the Crocin-L group, Crocin-H group and control group were all kept in a smoke-free environment. After 16 weeks of smoke exposure, crocin was administered intragastrically to the mice once daily for 4 weeks to the Crocin-L (20 mg/kg/day) and Crocin-H (60 mg/kg/day) groups (Dianat et al., 2018; Xie et al., 2019a). The Model and Control groups received an equivalent volume of 0.9% saline solution during the same period. After 20 weeks, mice were anesthetized with 2% pentobarbital sodium (1 mL/kg) intraperitoneally, blood was collected by puncturing the retroorbital venous plexus of mice, and lung tissues were immediately perfused for lung histological examination.

#### 2.6.4 General symptoms and signs in mice

After 7 days of adaptive feeding, the fur status, activity and mental status of mice in each group were observed and recorded. Starting from week 17, weight changes were recorded every 4 days.

#### 2.6.5 Lung histopathology detection

After euthanasia, lung tissues were fixed in 4% paraformaldehyde, dehydrated, embedded in paraffin, and cut into 4 μm serial sections. Sections were then stained with hematoxylin and eosin (H&E) for microscopic evaluation of morphological changes.

#### 2.6.6 ELISA determination

Blood samples were centrifuged at 3,000 × g for 10 min at 4 °C to separate serum, which was then stored at −80 °C until analysis. Serum levels of IL-6 and TNF-α were measured using commercially available ELISA kits according to the manufacturer’s protocols.

#### 2.6.7 Western blot

Total protein was extracted from cell or tissue samples using lysis buffer followed by centrifugation. Protein concentration was determined using a BCA protein assay kit. The protein samples were separated on 10% SDS-PAGE gels and transferred to PVDF membranes. After blocking with 5% non-fat milk, the membranes were incubated overnight at 4 °C with primary antibodies, using GAPDH as an internal control. The next day, the membranes were incubated with secondary antibodies for 1 h at 37 °C. Protein bands were visualized using an ECL kit, and the grayscale values of the Western blot bands were analyzed with ImageJ software.

### 2.7 Analytical statistics

All data were analyzed using GraphPad Prism 9 software. Data were analyzed for normality using Kruskal-Wallis test and Q-Q plots. Data in this study were all normally distributed and presented as mean ± standard error of the mean (S.E.M.). Comparisons between two groups were performed using a two-tailed unpaired t-test, and analysis of significance of differences between multiple groups was performed using one-way analysis of variance (ANOVA) and followed by Tukey post-hoc test. *P* < 0.05 was considered statistically significant.

## 3 Results

### 3.1 Identification of potential target genes of crocin against COPD via network analysis

#### 3.1.1 Crocin and COPD common target prediction

Using the PharmMapper and SEA databases, we screened 306 potential targets of crocin (MaxTC >0.4). Intersecting these with 10,353 COPD-related genes obtained from the GeneCards, OMIM, and TTD databases, we identified a total of 243 common targets (Figure 1B).

#### 3.1.2 Construction and analysis of PPI network

The PPI network constructed using the STRING database contains 242 nodes and 735 edges (confidence >0.7) (Figure 1C). 48 candidate targets (Figure 1D) were selected using Cytoscape topology analysis by screening with values above the median (DC > 5.00, BC > 152.48, CCY > 0.13, EC > 0.01, LAC >1.33). Further, the top 10 core targets were identified according to the most common parameter, Degree value: ALB, AKT1, EGFR, ESR1, CASP3, HSP90AA1, MMP9, PPARG, SRC, and IGF1 (node color from light to dark red indicates increasing Degree values) (Figure 1E).

#### 3.1.3 GO and KEGG pathway enrichment analysis

**FIGURE 2:**
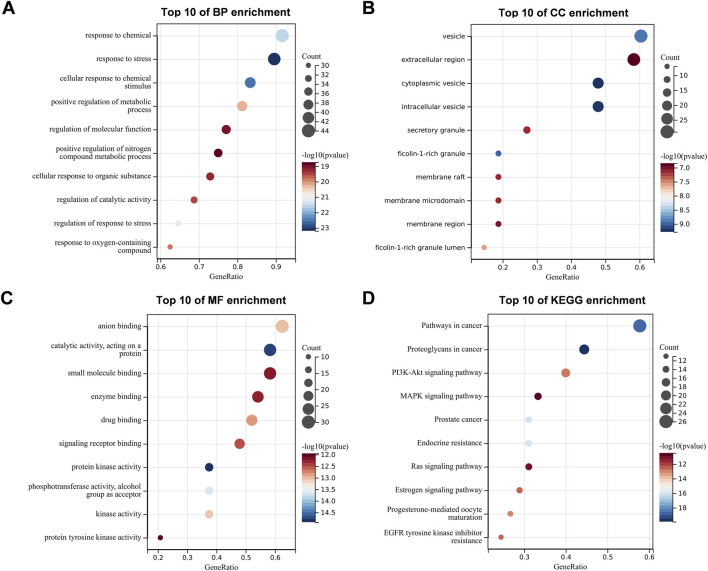
GO and KEGG analysis. **(A–C)** Top 10 enriched GO terms for 48 candidate targets. **(D)** Top 10 enriched KEGG pathways for 48 candidate targets. BP: biological process, CC: cellular component, MF: molecular function, *P* < 0.05. GO: Gene Ontology, KEGG: Kyoto Encyclopedia of Genes and Genomes, *P* < 0.05.

GO enrichment and KEGG enrichment analysis were performed on 48 candidate targets. GO enrichment analysis showed that the target genes of crocin in the treatment of COPD were associated with 2503 BPs, 134 CCs and 247 MFs (*P-*value <0.05 and FDR <0.1). The most significantly associated among the BP enrichments were response to stress, regulation of molecular function, positive regulation of nitrogen metabolic process, etc. (Figure 2A). The most significantly associated extracellular region, cytoplasmic vesicle, intracellular vesicle among others in CC (Figure 2B). MF analysis revealed that catalytic activity (acting on a protein), enzyme binding, protein kinase activity, and protein tyrosine kinase activity were correlated (Figure 2C). KEGG enrichment analysis suggests that crocin regulates the pathological process of COPD via signaling pathways that are closely related to such as PI3K-AKT, MAPK, Ras and Proteoglycans in cancer (Figure 2D; Supplementary Table S1).

### 3.2 Molecular docking

**FIGURE 3:**
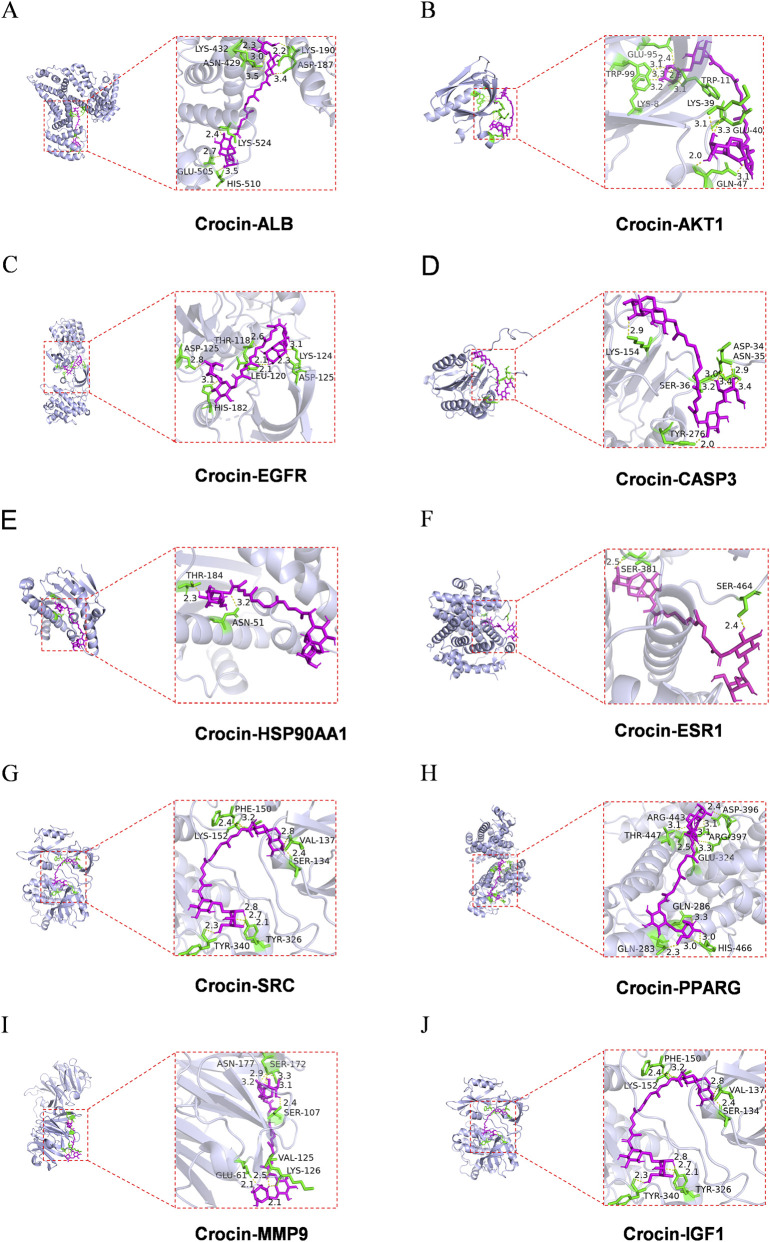
Pattern diagram of molecular docking. **(A)** Molecular docking of crocin and ALB. **(B)** Molecular docking of crocin and AKT1. **(C)** Molecular docking of crocin and EGFR. **(D)** Molecular docking of crocin and CASP3. **(E)** Molecular docking of crocin and HSP90AA1. **(F)** Molecular docking of crocin and ESR1. **(G)** Molecular docking of crocin and SRC. **(H)** Molecular docking of crocin and PPARG. **(I)** Molecular docking of crocin and MMP9. **(J)** Molecular docking of crocin and IGF1. The left side shows the 3D structure of the target protein, the red box represents the binding pocket structure, and the right side shows the enlarged display of the binding pocket. The rose part represents crocin, the green part represents the binding residue, the yellow dashed line represents the hydrogen bond, and the text part represents the residue name.

To investigate the interaction between crocin and the top 10 core targets, molecular docking was performed (Figure 3). Binding energy is used to evaluate the tightness of binding. The lower the binding free energy, the higher the likelihood of ligand binding to the receptor. A binding energy lower than −5 kcal/mol is generally considered indicative of effective binding. Our results showed that the binding energies of crocin to all core targets were ≤− 7.7 kcal/mol (Table 1). Among them, ALB (PDB: 1BJ5) and SRC (PDB: 1FMK) exhibited the lowest binding energy to crocin (−9.6 kcal/mol). Other targets, including AKT1 and EGFR (both at −8.4 kcal/mol), also demonstrated stable binding. These results collectively indicate that crocin has a strong binding affinity to the core targets associated with COPD.

**TABLE 1:** Binding energy of crocin to top 10 core targets.

Target	PDB ID	Binding energy (kcal/mol)
ALB	1BJ5	−9.6
AKT1	1H10	−8.4
EGFR	4HZR	−8.4
CASP3	2CJY	−7.7
HSP90AA1	3O0I	−8.0
ESR1	6PSJ	−8.5
SRC	1FMK	−9.6
PPARG	8B94	−9.5
MMP9	1ITV	−9.0
IGF1	4XLV	−9.3

### 3.3 Molecular dynamics simulation

**FIGURE 4:**
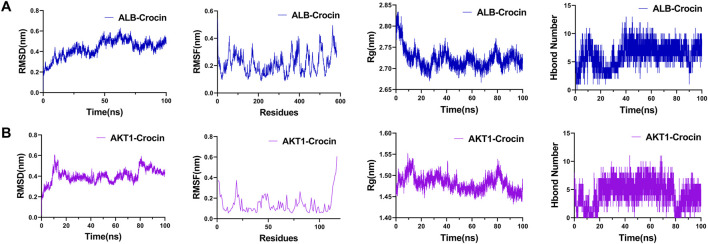
Molecular dynamics (MD) simulation results. **(A)** ALB-crocin. **(B)** AKT1-crocin. RMSD: root mean square deviation of the complex, RMSF: root mean square fluctuation in the complex, Rg: radius of gyration of the complex, Hbond number: number of hydrogen bonds of the complex.

To further assess the possible target of crocin in improving COPD, molecular dynamics simulations of ALB and AKT1 were performed for 100 ns to reveal the stability of protein-ligand (ALB-crocin, AKT1-crocin) interactions. Root mean square deviation (RMSD) values were used to assess whether the simulated system had reached a steady state, and RMSD values below 1 nm indicated the relative stability of protein-ligand interactions in physiological environments (Sarker et al., 2023). For the ALB-crocin complex structure, the RMSD rapidly stabilized at 0.4 ± 0.2 nm after 12 ns. Root mean square fluctuation (RMSF) showed that domain II (residues 200–300) fluctuated the least (<0.3 nm) and the number of hydrogen bonds remained stable at 7 ± 1 (Figure 4A) (Park et al., 2021). The RMSD of the AKT1-crocin complex structure was stabilized at 0.4 ± 0.1 nm after 14 ns, the catalytic domain (residues 150–250) RMSF was <0.5 nm, and the number of hydrogen bonds was maintained at 6 ± 1 (Figure 4B) (Truebestein et al., 2021). Radius of gyration (Rg) analysis showed that the complex structure was compact (ALB: 2.7 ± 0.1 nm; AKT1:1.48 ± 0.05 nm), indicating that the complex structure was stable.

### 3.4 Crocin directly acting on AKT1

**FIGURE 5:**
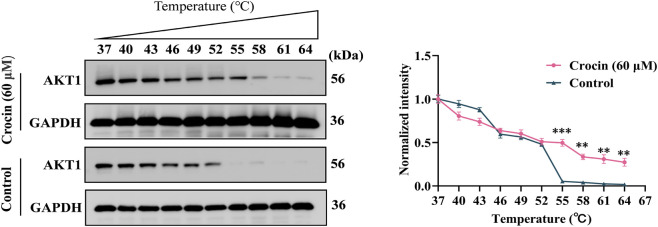
Cellular Thermal Shift Assay of AKT-1 in BEAS-2B cells, cells treated with or without crocin (60 µM) for 24 h.

To assess if crocin directly interacts with AKT1, we performed a CETSA. Since a prior CCK-8 assay had confirmed that crocin concentrations up to 60 μM were non-cytotoxic to BEAS-2B cells (Supplementary Figure S1), we selected 60 μM for CETSA. After 24 h treatment, crocin significantly enhanced the thermal stability of AKT1. AKT1 protein rapidly diminishes as the temperature increases from 37 °C to 64 °C, almost disappearing completely after 52 °C. This indicates that AKT1 protein denatures and precipitates under high temperature, making it undetectable in the cell lysate. In the crocin-treated group (Crocin, 60 µM), the intensity of the AKT1 protein band at high temperatures (e.g., 55 °C, 58 °C, 61 °C) is significantly higher than that of the control group at the same temperatures. The internal reference GAPDH remains stable under all conditions, ensuring the comparability of the results. This visually demonstrates that AKT1 protein in crocin-treated cells is more tolerant to high temperature, while the control protein GAPDH is unaffected by temperature changes, indicating that the binding between AKT1 and crocin is relatively specific (Figure 5).

### 3.5 Crocin improves COPD in mice by downregulating p-AKT1

**FIGURE 6:**
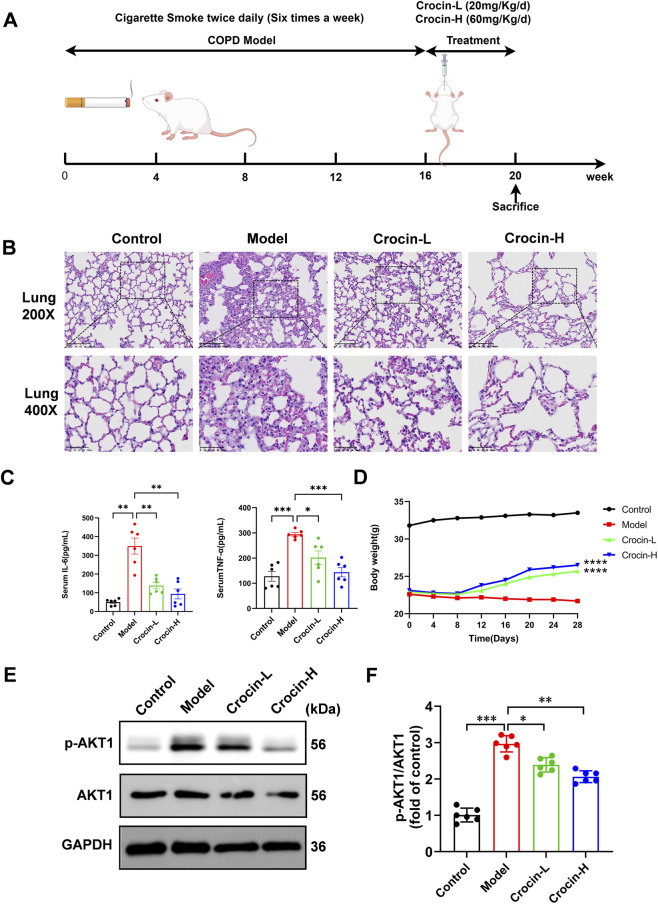
*In vivo* experimental validation of crocin in improving COPD mouse models. **(A)** COPD modeling process. **(B)** Representative HE staining (200× and 400×) for mouse lung tissue. **(C)** ELISA to determine IL-6 and TNF-α in mouse serum (N = 6). **(D)** Body weight of mice in each group during crocin gavage after the end of modeling (16 weeks later, N = 6). **(E)** p-AKT, AKT, and GAPDH protein bands were detected by Western blot (N = 6). **(F)** p-AKT/AKT protein ratios (N = 6) (*p < 0.05, **p < 0.01, ***p < 0.001) vs. Model.

First, mouse model of COPD was established by smoking (Figure 6A). Mice in the Model group showed unkempt fur, yellow discoloration and increased shedding after smoking, and gradually developed symptoms such as unresponsiveness, slowness of movement, occasional agitation, and tendency to huddle in the late stage of modeling. In contrast, mice in the control group had healthy fur, bright color, and no yellowing, dryness, or disorganization. At the same time, HE staining showed that some alveolar walls of mice in the Model group collapsed, the alveoli irregularly expanded and fused with each other, and formation of bullae, and significant focal inflammatory cell infiltration and massive fibrous hyperplasia were observed in the pulmonary interstitium. However, in the Control group, the alveoli were morphologically normal and structurally intact, with no significant thickening of smooth muscle or inflammatory infiltration observed (Figure 6B). In addition, further ELISA revealed that IL-6 and TNF-α levels in the serum of Model group increased significantly compared with the Control group (Figure 6C). These results indicate that a mouse model of COPD has been successfully established via smoking.

Secondly, HE staining and ELISA showed that after treatment with crocin, a small amount of fibrous hyperplasia and inflammatory cell infiltration were observed in the lung tissues of mice in the Crocin-H and Crocin-L groups, and no additional significant lesions were noted, and these changes were significantly improved compared with the model group. Meanwhile, the serum levels of IL-6 and TNF-α were significantly decreased in the Crocin-H and Crocin-L groups, suggesting that crocin effectively reduced the inflammatory response in lung tissue in COPD and provided histological improvement. In addition, the body weight of mice in the Crocin-L and Crocin-H groups increased significantly after 12 days of improvement, compared with the body weight decrease in the Model group (Figure 6D), and the difference was statistically significant (*P* < 0.0001).

To further explore the potential mechanism, we evaluated the expression of AKT1—a potential target identified in our earlier analyses—in the lung tissues. Western blot analysis revealed that crocin treatment markedly reduced the p-AKT1/AKT1 ratio, indicating that its anti-inflammatory and lung-protective effects are, at least in part, mediated through inhibition of the AKT1 pathway (Figures 6E,F).

## 4 Discussion

This study integrated predictive network analysis and computational simulations to identify potential therapeutic targets for crocin in COPD, and then conducted experiments to assess these predictions, thereby exploring the underlying mechanisms and providing a basis for early clinical diagnosis, treatment, and prevention. The main findings indicate that, guided by computational predictions, AKT1 was prioritized as a candidate target. Subsequent experimental evaluation using the CETSA revealed a direct interaction between crocin and AKT1. *In vivo* results from a smoke-induced COPD mouse model demonstrated that crocin effectively alleviated pathological lung injury and systemic inflammation.

To predict the multi-target potential of crocin in COPD, network analysis identified 48 common targets, with 10 core targets screened based on Degree values: ALB, AKT1, EGFR, ESR1, CASP3, HSP90AA1, MMP9, PPARG, SRC, and IGF1 (Figure 1E). Among these, proteins like AKT1, SRC, EGFR, and HSP90AA1 function as key signaling mediators amplifying inflammatory responses via downstream pathways such as NF-κB and MAPK (Di Lorenzo et al., 2009; Ren et al., 2020; Zhang et al., 2022; Zhang et al., 2023). MMP9 and CASP3 act as inflammatory effectors involved in extracellular matrix degradation and programmed cell death, respectively (Liang et al., 2018; Wang et al., 2025). Meanwhile, ESR1 and PPARG serve as endogenous negative regulators suppressing excessive inflammation (Cephus et al., 2021; Larson-Casey et al., 2023). Functional enrichment analysis indicated that these targets are involved in protein binding and extracellular localization, and are significantly associated with PI3K-AKT, Ras, and MAPK signaling pathways.

To further evaluate potential interactions, computational simulations were performed. Molecular docking indicated strong binding affinities between crocin and all ten candidates (binding energies <−7.7 kcal/mol). Molecular dynamics simulations demonstrated stable interactions for ALB and AKT1. Specifically, for the AKT1-crocin complex, the RMSD stabilized at 0.4 ± 0.1 nm after 14 ns, the RMSF of catalytic domain was <0.5 nm, and hydrogen bonds remained at 6 ± 1 (Figure 4B) (Truebestein et al., 2021). Although ALB plays an important pharmacokinetic role in inflammatory disease (Sleep, 2015), it is not expressed in pulmonary epithelial cells; therefore, subsequent experimental investigation focused on AKT1, which was present in all top enriched pathways (Supplementary Table S1).

We then assessed target engagement using CETSA. Our data showed that treatment with crocin (60 μM, 24 h) significantly enhanced the thermal stability of AKT1, suggesting that crocin directly binds to and stabilizes this target in live BEAS-2B cells. These findings provide preliminary support for the hypothesis that AKT1 may be a potential target through which crocin alleviates COPD. On this basis, we further examined the effects of crocin in an animal model of COPD and explored its possible mechanism related to AKT1.

AKT1, a central effector in the PI3K-AKT pathway, plays a pivotal role in regulating cell survival, proliferation, metabolism, and inflammatory responses (Xie et al., 2019a; Lin X. et al., 2022; Liu et al., 2023). Previous research has demonstrated that cigarette smoke activates the PI3K-AKT/NF-κB axis, promoting AKT1 phosphorylation, NF-κB nuclear translocation, and pro-inflammatory cytokine release (Xie et al., 2019b). In the present study, crocin intervention not only significantly ameliorated weight loss and pulmonary pathological injury in COPD mice and reduced serum levels of IL-6 and TNF-α, but Western blot analysis also showed that it downregulated the p-AKT1/AKT1 ratio in lung tissue. These *in vivo* data are consistent with the cellular evidence of target engagement. We propose that crocin binds to and stabilizes AKT1, thereby inhibiting its phosphorylation and downstream NF-κB activation. This mechanism reduces TNF-α and IL-6 production, ameliorating COPD pathology via an AKT1-mediated anti-inflammatory pathway that warrants further study.

We acknowledge several limitations in this study. COPD mouse model assessment relied on histopathology, serum cytokines, and disease phenotypes; pulmonary function testing was not performed due to constraints. The lack of a positive control group in animal experiments is also recognized. Future studies will incorporate appropriate positive controls for benchmarking. It is particularly important to emphasize that crocin’s extended conjugated polyene system shares structural features with PAINS (Magalhães et al., 2021), which can produce false positives via non-specific mechanisms. Thus, high docking-affinity predictions require cautious interpretation. However, our combined approach mitigates this concern: crocin’s efficacy was observed in a complex physiological COPD model less susceptible to PAINS artifacts than simplified *in vitro* systems. Moreover, CETSA results in live cells provide physiologically relevant evidence less prone to non-specific interference (Bolz et al., 2021). Future studies using target-specific knockout models or orthogonal binding assays will further confirm the crocin-AKT1 interaction specificity.

## 5 Conclusion

In summary, our findings suggest that crocin alleviates disease symptoms by stabilizing AKT1 and reducing the level of p-AKT1 in mouse COPD model, supporting its potential as a therapeutic candidate for COPD. We have not only further confirmed its promise as a precision treatment strategy but also established a novel framework for identifying context-specific therapeutic targets through the integration of computational prediction and experimental evaluation. Future research should focus on conducting clinical trials to systematically evaluate the efficacy, safety, and pharmacokinetic profile of crocin in COPD patients, thereby providing a new candidate drug for COPD treatment.

## Data Availability

The raw data supporting the conclusions of this article will be made available by the authors, without undue reservation.

## Supplementary material

SUPPLEMENTARY FIGURE S1 Cell viability of crocin in BEAS-2B cells, cells treated with or without crocin (60 µM) for 24 h.
