# Supplementary material for: Crocin protects against smoke-induced chronic obstructive pulmonary disease by regulating AKT1
Source: Front Pharmacol. 2026 Feb 9;17:1687752. doi: 10.3389/fphar.2026.1687752 (PMC12926455; doi:10.3389/fphar.2026.1687752)
Supplement: Supplementary file 1 [file Table1.docx]

**Supplementary Table 1:Top 10 of KEGG enrichment**

| ID | Description | GeneRatio | pvalue | p.adjust | geneID |
| --- | --- | --- | --- | --- | --- |
| hsa05205 | Proteoglycans in cancer | 20/48 | 1.19E-20 | 2.37E-18 | MMP2/IGF1/MAPK14/PLAU/KDR/FGF2/IGF1R/MET/AKT1/MAPK1/RHOA/PRKACA/PTPN11/MDM2/MAP2K1/EGFR/SRC/CASP3/MMP9/ESR1 |
| hsa05200 | Pathways in cancer | 26/48 | 1.21E-19 | 1.21E-17 | MMP2/IGF1/FGF2/MAPK8/IGF1R/MET/CCNA2/AR/AKT1/RXRA/JAK2/PPARG/MAPK1/RHOA/PRKACA/F2/GSTP1/MDM2/MAP2K1/EGFR/CDK2/HSP90AA1/CASP3/MMP9/IL2/ESR1 |
| hsa05215 | Prostate cancer | 14/48 | 8.02E-17 | 4.66E-15 | IGF1/PLAU/IGF1R/MMP3/AR/AKT1/MAPK1/GSTP1/MDM2/MAP2K1/EGFR/CDK2/HSP90AA1/MMP9 |
| hsa01522 | Endocrine resistance | 14/48 | 9.32E-17 | 4.66E-15 | MMP2/IGF1/MAPK14/MAPK8/IGF1R/AKT1/MAPK1/PRKACA/MDM2/MAP2K1/EGFR/SRC/MMP9/ESR1 |
| hsa04914 | Progesterone-mediated oocyte maturation | 12/48 | 1.34E-13 | 5.36E-12 | IGF1/MAPK14/MAPK8/IGF1R/CCNA2/AKT1/MAPK1/PRKACA/MAP2K1/CDK2/PGR/HSP90AA1 |
| hsa04151 | PI3K-Akt signaling pathway | 18/48 | 1.91E-13 | 6.36E-12 | SYK/IGF1/KDR/FGF2/IGF1R/MET/AKT1/RXRA/JAK2/MAPK1/EPHA2/NOS3/MDM2/MAP2K1/EGFR/CDK2/HSP90AA1/IL2 |
| hsa04915 | Estrogen signaling pathway | 13/48 | 3.53E-13 | 9.23E-12 | MMP2/AKT1/MAPK1/HSPA8/PRKACA/NOS3/MAP2K1/EGFR/SRC/PGR/HSP90AA1/MMP9/ESR1 |
| hsa01521 | EGFR tyrosine kinase inhibitor resistance | 11/48 | 3.69E-13 | 9.23E-12 | IGF1/KDR/FGF2/IGF1R/MET/AKT1/JAK2/MAPK1/MAP2K1/EGFR/SRC |
| hsa04014 | Ras signaling pathway | 14/48 | 1.74E-11 | 3.88E-10 | IGF1/KDR/FGF2/MAPK8/IGF1R/MET/AKT1/MAPK1/RHOA/EPHA2/PRKACA/PTPN11/MAP2K1/EGFR |
| hsa04010 | MAPK signaling pathway | 15/48 | 3.29E-11 | 6.58E-10 | IGF1/MAPK14/KDR/FGF2/MAPK8/IGF1R/MET/AKT1/MAPK1/EPHA2/HSPA8/PRKACA/MAP2K1/EGFR/CASP3 |
