# Supplementary figures and images for: Crocin protects against smoke-induced chronic obstructive pulmonary disease by regulating AKT1
Source: Front Pharmacol. 2026 Feb 9;17:1687752. doi: 10.3389/fphar.2026.1687752 (PMC12926455; doi:10.3389/fphar.2026.1687752)

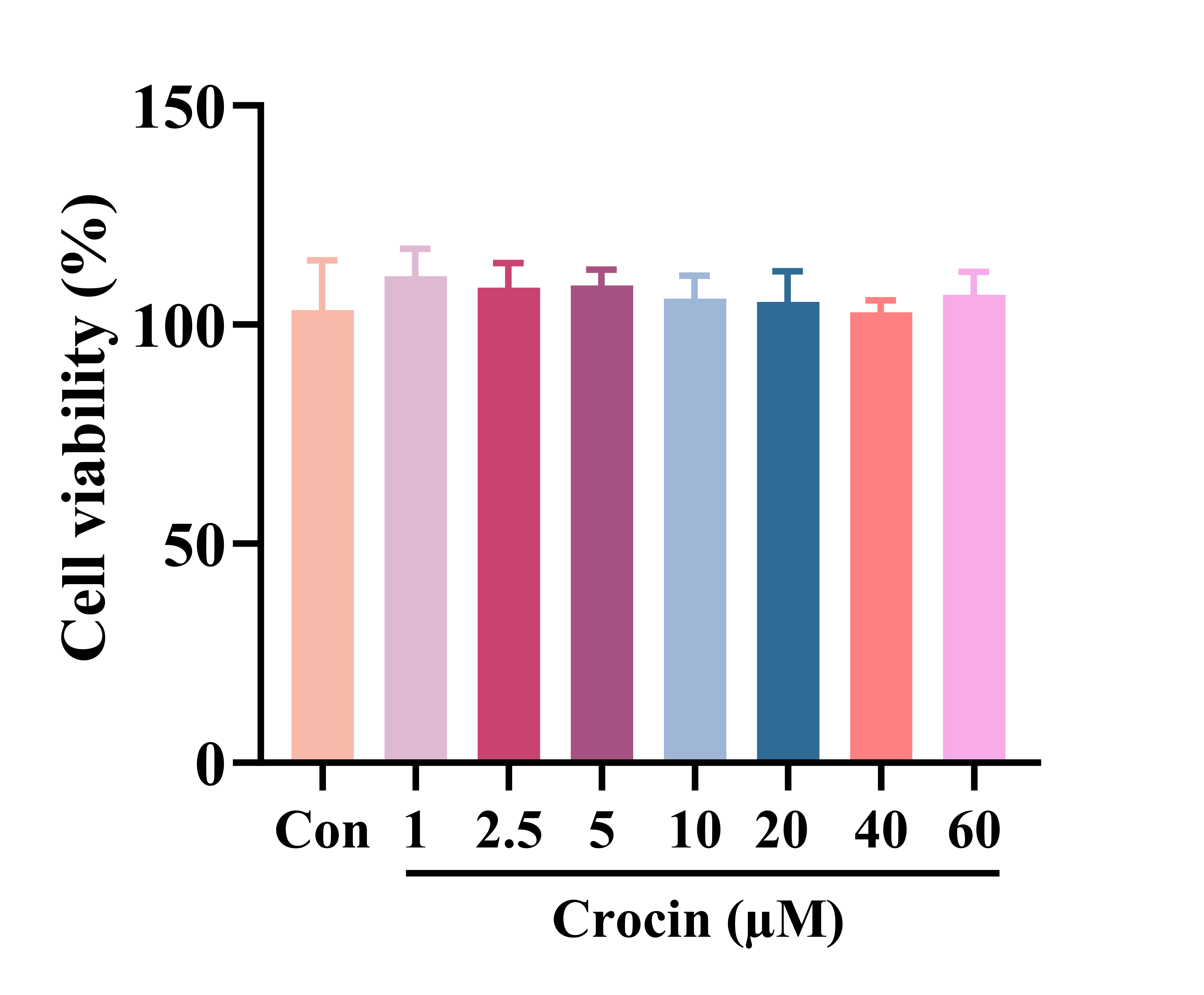

Supplement: Supplementary file 2 [file Image1.tif]
